# COVID-19: losing battles or winning the war?

**DOI:** 10.1186/s42522-020-00019-2

**Published:** 2020-05-19

**Authors:** Leslie A. Reperant, Albert D. M. E. Osterhaus

**Affiliations:** 1 Artemis One Health Research Foundation, Delft, The Netherlands; 2 University of Veterinary Medicine Hannover, Hannover, Germany

“Every battle is won or lost before it is even fought.” This statement is attributed to Sun Tzu in the Art of War [1], an ancient Chinese military treatise dated from the fifth century BC. It highlights the importance of preparation, positioning and planning before engaging in battle. The emergence of the COVID-19 pandemic painfully revealed how many countries had embarked on a battle that was lost even long before the new coronavirus had reached their borders. More strikingly, most have persisted in this defeatist attitude as the crisis deepened, failing to re-direct their strategy. Living with SARS-CoV-2 by returning as closely as possible to “business as usual” is far from winning the battle. It rather looks like “giving in to the enemy”, while vaccine and drug development efforts only feed the dearest hope for a successful way out.

## Preparation

The emergence of a new viral pandemic was, is and remains a matter of when, rather than if, even today amidst a raging pandemic. Among the prime candidates are those caused by influenza viruses that originate from animals. The SARS and MERS outbreaks in the past two decades highlighted the threat posed by animal coronaviruses before the unprecedented COVID-19 assault started. But we should not forget that members of other virus families affecting the animal kingdom can also be quite successful at jumping host species, like paramyxoviruses, hantaviruses, filoviruses, bunyaviruses, flaviviruses, and more. The number of newly emerging viruses in the human population, most of which originate from animals, has dramatically increased in the past decades. However, this exchange of pathogens at the human-animal interface is not new [2]. Childhood diseases, such as smallpox, mumps and measles, have been acquired up to thousands of years ago by transmission of their ancestral viruses from domesticated livestock. In the past centuries, influenza- and metapneumoviruses have been transmitted from birds to humans and have become established as recurring seasonal scourges. More recently, countless reports of novel viral disease emergence events have made the news, revealing a most worrying trend. These include the AIDS pandemic caused by HIV, hemorrhagic fever outbreaks caused by arena-, hanta- flavi- and filoviruses, pneumonia and viral encephalitis caused by Nipah and Hendra viruses, debilitating arthralgia in people with Chikungunya, or even more unexpected ailments such as microencephaly in newborns with Zika. This trend is mirrored by a similar increase in viral outbreaks among wild and domestic animal species worldwide, threatening and often decimating their populations. It is fueled by dramatically accumulating anthropogenic changes of our planet, including relentless urbanization and industrialization, natural habitat destruction, global trade and travel, collectively making the current geological epoch, the “Anthropocene” [3]. These global changes lead to increased human-to-human contacts even across large geographical distances and to increased human-animal contacts, involving both domestic and wild species, in often mixing populations. As these anthropogenic changes are largely determined by human behavior and therefore generally hard to influence, new viral threats will continue to emerge, ever more frequently. This warning has been raised for many decades now, and re-iterated upon each novel emergence, like Nipah, avian and pandemic influenza, SARS, MERS, Ebola, Zika, all calling for epidemic and pandemic preparedness. Were we prepared for a pandemic like that of COVID-19? **No, we were not.**

## Positioning

The new coronavirus, SARS-CoV-2, causing COVID-19, emerged in Wuhan, China in late 2019 [4]. Within a few weeks, it demonstrated efficient human-to-human transmission with a basic reproduction number (R_0_) initially estimated between 1.4 and 3.8 [5–8]. On 26 January 2020, China’s National Health Commission Minister Ma Xiaowei informed the public and the world in a press conference that infected individuals can spread the virus before they develop symptoms [9]. Control measures against a transmissible virus that can be spread ‘pre-symptoms’ are bound to be different from those against a transmissible virus that spreads ‘post-symptoms’. Screening for fever and other signs of disease in order to identify, test and quarantine infected individuals will be sufficient to interrupt chains of transmission of a ‘post-symptom-spreading’ virus (as with the SARS coronavirus) but will unlikely be sufficient for a ‘pre-symptom-spreading’ virus [10]. Likewise, control measures against a highly transmissible virus are bound to be different during the phase of importation of the virus from its source outbreak than upon widespread regional community transmission. While closing borders to all travelers coming from epicenters of infection or quarantining them may be of little practicality upon widespread community transmission, it is an essential defense to prevent virus introduction during the phase of importation. Interestingly, upon introduction of an emerging foreign animal disease in a previously naïve country, the widely accepted control strategy is containment and elimination [11]. The early phase of importation characterized by limited local transmission offers a short window of opportunity for successful control, provided strict and not half-hearted containment measures are applied to hit the emerging pathogen hard. These measures are successfully applied in well-defined control and surveillance zones with firm restrictions of contact and movement of animals independent of their infection status. Was a similar strategy adopted by most governments during the initial importation phase of the COVID-19 pandemic? **No, it was not.**

## Elimination vs mitigation

Mitigation or damage control was the preferred position of most governments. Such a position may seem alluring since less strict and less aggressive measures are applied, limiting their associated costs. However, this perceived advantage is malignantly deceiving as the applied control measures carry a non-negligible risk to be insufficient at preventing widespread regional community transmission. It eventually calls for increasingly more resources as the virus continues to circulate, further spreads, and imposes rising morbidity and mortality burdens. This approach resulted in the first waves of the COVID-19 pandemic and necessitated extensive lockdowns in many countries. The largely successful elimination strategy practiced by some Asian countries that had experienced SARS and/or MERS outbreaks in the past, sharply contrast with the shortcomings of mitigation strategies. Even today, the welcome decreasing trends in the number of new cases in most countries hit by a first wave entice both governments and the public to relax control measures relying on limited evidenced-based criteria and fueled by short-term economic and societal concerns. However, a most likely consequence will be the spark of new chains of transmission and new epidemic flares or waves. This will result in lengthening the outbreak, further increasing economic and societal costs, let alone human suffering. In this light, it is interesting to note that socio-economic analyses of the impact of emerging foreign animal disease control clearly show that the most (cost-)effective strategies aim at reducing the length of the outbreak, which is achieved through elimination and not mitigation [12].

Models have hinted at the need for implementing an on-and-off lock-down strategy against COVID-19 until vaccines are available for the world population [13, 14]. Here also, the apparent alluring advantages of imposing and lifting mitigating measures to mainly avoid overwhelming healthcare needs while keeping life disruption to the minimum possible are most deceiving. Such a proposal is likewise fueled by blind-folded short-term economic and societal concerns, while insensitive to long-term human health and socio-economic costs. As currently experienced in many countries, lifting mitigation measures without experiencing an epidemic rebound, calls for a delicate implementation strategy that requires costly adjustments of common practice in many sectors. For example, public transport, shops and restaurants should function at dramatically reduced capacity, for an as yet unknown period of time. The most vulnerable population, i.e., people over 70 and people with underlying chronic conditions, will need to remain isolated, with substantial health and psychological consequences. In the meantime, all hopes are channeled towards future COVID-19 vaccines. However, the road to safe and effective vaccines is fraught with multiple developmental and regulatory hurdles and uncertain timelines, while their equitable distribution among those who need them most is not guaranteed. Be it as it may, the expected advent of a vaccine cannot serve as an escape route from taking all necessary measures to resolve the crisis today. Most countries are increasingly entangled in largely unsustainable mitigation strategies. In view of their inherent pitfalls, is pursuing mitigation rather than embarking on elimination a rational position to emerge from this crisis? **No, it is not.**

## Planning

Planning supports both preparation and positioning efforts. It is done preferably in ‘peace time’, for example by stockpiling essential assets, implementing platform technologies that can rapidly provide specific diagnostics, therapeutic and vaccine leads upon a viral disease emergence, and elaborating comprehensive outbreak response plans. Although planning in ‘peace time’ may seem expensive, the investment is manifestly justified as an insurance policy for pandemic events with draconic economic and societal consequences that rapidly will dwarf these costs. The blatant revelation of the limited capacity of healthcare systems to cope with an epidemic surge of severe cases demonstrated tragic planning deficits. The capacity for rapidly producing and deploying diagnostic tests and critical materials, well trained personnel, and infrastructure, remains a challenge. Even today, while lifting strategies are being outlined and planned, the most effective test-trace-quarantine strategy is erroneously considered secondary at best by many governments. Lifting mitigation measures with poor and non-evidenced-based planning appears to be politically driven by economic and societal pressures, bordering a lack of courage and determination. Can this battle be won without a thorough tactical response built on scientific and public health intelligence? **No, it cannot.**

## Winning the war

The positioning of most governmental strategies needs to be resolutely realigned with elimination and not mitigation goals, while continuing to support all efforts aiming at vaccine and drug development. The lockdown and restraint measures put in place in most countries will eventually flatten the epidemic curve to sufficiently low levels that create a new opportunity for successful elimination. The effective coordination of diagnostic testing, contact tracing, and strict quarantining of all contacts of confirmed cases must be ramped up during lockdown, before measures are lifted, and movement and contact have increased and intensified. Only a strategic coordination of this three-step approach (test, trace, quarantine) as maximal social distancing measures are in place, in combination with fast, efficient and scaled-up digital contact tracing [15], will fully leverage all the costly efforts that have been made to decrease SARS-CoV-2 spread. This will enable governments to outpace rather than chase the virus, towards its successful elimination. It will allow transitioning from countrywide lockdowns to targeted quarantines. Unless governments maintain travel restrictions from and to countries with continued circulation of SARS-CoV-2, an international cooperative and coordinated response aligned on a shared elimination goal is imperative. Beyond COVID-19, pandemic preparedness against future emerging viral threats must be genuinely and purposefully addressed, likewise within an international cooperative and coordinated framework, engaging all sectors concerned. Lastly, major societal changes are unavoidable to drastically limit the impact of mankind on the global environment and ecosystems, to promote the safeguarding and restoration of natural habitats while striving at global equalities to all human fundamental rights. Unrestrained economic growth must be reconsidered to build on more balanced and sustainable socio-economic principles. This pandemic is giving us a wake-up call to unambiguously fight to interrupt its spread and to conscientiously prepare for future pandemics, while offering a truly unique opportunity to alter the course of mankind’s ecological footprint on the planet. **Let’s not waste it.**
